# Development of a Brief Online Mindfulness-Based Intervention to Reduce Patient Anxiety Before a First-Time Screening Colonoscopy

**DOI:** 10.1007/s13187-025-02608-z

**Published:** 2025-03-31

**Authors:** Brent Emerson, Paul L. Reiter, Maryanna Klatt, Darrell M. Gray, Hisham Hussan, Subhankar Chakraborty, Mira L. Katz

**Affiliations:** 1 Division of Health Behavior and Health Promotion, College of Public Health, The Ohio State University, Columbus, OH USA; 2 Comprehensive Cancer Center, The Ohio State University, Columbus, OH USA; 3 Center for Integrative Health, Department of Family and Community Medicine, College Medicine, The Ohio State University, Columbus, OH USA; 4 Department of Internal Medicine, College of Medicine, The Ohio State University, Columbus, OH USA

**Keywords:** Colorectal cancer, Screening, Mindfulness, Mindfulness-based intervention

## Abstract

To describe the development of an online mindfulness-based intervention (MBI) to reduce anxiety before a first-time screening colonoscopy among average-risk patients. A qualitative study used an iterative process guided by health behavior and mindfulness theories and feedback from a convenience sample of patients, endoscopy medical staff, and community members. Patient and medical staff (*n* = 18) were included in formative interviews (30–45 min), eight helped during intervention development sessions (15–90 min), and four community members reviewed the MBI in individual sessions (60 min). Interviews and sessions were recorded, transcribed verbatim, and analyzed using NVivo qualitative data software. Two themes emerged from the study: (1) both patients and medical staff reported that average-risk patients have pre-procedural anxiety before a first-time screening colonoscopy, and (2) using stakeholder-engaged strategies in an iterative process with both patients and medical staff is important so the developed intervention is acceptable to the priority population and to ensure medical accuracy and avoid disruption of workflow. Using an iterative process with key stakeholders is essential to develop interventions that are feasible and acceptable. The MBI developed through this process is being compared to usual care in a pilot randomized controlled trial to determine intervention feasibility and patient acceptability and to collect preliminary efficacy data. If efficacious, the developed MBI has the potential to reduce pre-procedural anxiety which may improve patient behaviors (e.g., bowel prep adherence and quality), patient satisfaction, and clinic workflow by reducing cancellation/no-shows, the amount of sedation required, and procedural time.

## Introduction

Colorectal cancer (CRC) is the second most common cancer, and the second leading cause of cancer-related deaths among males and females in the United States (U.S.) with an estimated 152,810 new cases and 53,010 deaths in 2024 [1]. CRC screening is cost-effective in reducing CRC incidence and mortality rates [2, 3]. The American Cancer Society (ACS) recommends average-risk adults ages 45–75 screen for CRC by high-sensitivity stool-based tests (e.g., fecal immunochemical test every year) or a direct visual examination (e.g., colonoscopy every 10 years) [4]. In 2021, only 58% of age-eligible adults were up-to-date with recommended CRC screening [5]. Because of the low screening prevalence, it is important to develop interventions to increase CRC screening.

Colonoscopy is considered the gold standard for screening as this procedure may prevent CRC through the removal of polyps and/or identify CRC in early stages when treatment is more successful [6]. Patient-reported barriers include anxiety associated with the bowel prep process and the procedure’s invasiveness [7, 8]. It is important to find ways to reduce pre-procedural anxiety since it may affect bowel prep adherence and quality, satisfaction with care, and intention for future CRC screening and may increase cancellation or no-shows, the amount of sedation required, and procedure time [7, 9–12]. Previous research to reduce patient anxiety before a colonoscopy has used educational materials, patient navigation, music, breathing exercises, guided imagery, and aromatherapy [13–17]; however, the use of mindfulness has not been explored for this purpose.

Mindfulness is a form of meditation that focuses on staying within the present moment to reduce anxiety [18]. A systematic review and meta-analysis of research among cancer patients and survivors supports mindfulness-based interventions (MBIs) to reduce anxiety, depression, fatigue, stress, and improve quality of life [19]. However, a critique of MBI research identified the lack of integration of mindfulness theory and transparency about MBI content development [20].

To address the limitations of previous studies that lacked information about MBI development and content, and the fact that MBIs have not been used to reduce patient anxiety prior to a first-time colonoscopy, this qualitative study describes the iterative stakeholder-engaged strategies used to develop a theoretically based brief online MBI to reduce anxiety before a first-time screening colonoscopy among average-risk patients.

## Methods

### Theoretical Framework

We developed a patient-level MBI to reduce pre-colonoscopy anxiety that included two components. The first component provided CRC screening educational information guided by the Protection Motivation Theory (PMT) [21], addressed common screening barriers, and sought to improve patients’ self-efficacy to complete the bowel prep and scheduled colonoscopy. The second component included mindfulness information and specific meditations guided by the Monitor and Acceptance Theory (MAT) [22, 23] aimed to increase attention and acceptance of negative emotions associated with completing screening.

### MBI Development

The MBI was developed from December 2020 to February 2023. We used an iterative process with stakeholders to develop the MBI that included formative interviews, content development, intervention development sessions, and review sessions. An interview guide was used to conduct the interviews and to facilitate the development and review sessions. Interviews and sessions were recorded, transcribed verbatim, and analyzed using NVivo qualitative software. The Institutional Review Board at The Ohio State University approved the study.

#### Formative Interviews

Formative interviews were conducted among average-risk patients after completing a first-time screening colonoscopy and with endoscopy medical staff (December 2020–November 2021). The interviews lasted 30–45 min and were conducted by telephone or videoconferencing to obtain patient and medical staff perspectives about the acceptability, feasibility, and the content and format to include in a MBI to reduce patient anxiety. Patients were eligible if they were ages 45–75, at average-risk, completed a first-time screening colonoscopy in the past week, did not have a cancer diagnosis, were able to speak English, and had a working phone or Internet connection. Medical staff were eligible if they were a gastroenterologist or nurse and worked in the endoscopy suite and interacted with patients undergoing a colonoscopy. A convenience sample of potentially eligible patients and endoscopy medical staff was identified by practicing gastroenterologists and was contacted to confirm eligibility and interest in participation and to obtain verbal informed consent which included permission to record the interview.

Among 12 patients, nine reported a willingness to participate in a MBI while in the waiting room before their colonoscopy and that they would have benefited from a MBI. Patients recommended that mindfulness meditations be brief (5 to 10 min), feature individuals from different races and ethnicities, and include nature-oriented backgrounds. Furthermore, patients suggested the MBI include educational information about the importance of CRC screening and the benefits of mindfulness to reduce anxiety.

Medical staff described the typical first-time screening colonoscopy patient as anxious and thought that a MBI may potentially benefit patients. However, medical staff also expressed concerns that a MBI in the endoscopy suite may disrupt workflow and interrupt their communication with patients. Therefore, they suggested that patients arrive 15 min before their scheduled arrival time for their colonoscopy to complete MBI activities in the waiting room prior to being admitted to the endoscopy suite.

#### MBI Content Development

Incorporating perspectives from patients, endoscopy medical staff, and guided by behavior and mindfulness theories, the study team developed MBI content, planned how the MBI would be implemented, and determined outcomes to be measured during evaluation. The developed MBI initially included beginning intervention activities 5 days before the scheduled colonoscopy in alignment with the start of recommended dietary changes before a screening colonoscopy, being delivered online for patient convenience, a landing page to brand the study’s identity by connecting patients to study content and activities, daily CRC screening and mindfulness educational infographics and meditations to improve engagement, and incorporating a brief meditation in the waiting room before the colonoscopy to mitigate impact on staff workflow.

The initial versions of the educational infographics were created and included five or six messages per infographic. The CRC screening infographics included messages that addressed PMT constructs, and the mindfulness infographics addressed common misconceptions of mindfulness and highlighted benefits of mindfulness practice. A study team infographic was developed to gain trust among patients and included photographs of team members with a brief description of their role in the study.

The content of the daily mindfulness meditation scripts incorporated feedback obtained during the interviews and was developed with guidance from a study team member certified in mindfulness meditation. We developed the meditation background (visual and audio) based on patient recommendations including nature-oriented photos (e.g., beach, stream), nature-oriented audio (e.g., waves, birds) and piano music for review during the next step of the process. In addition, due to the diversity of the patients and their feedback during the interviews, a diverse sample of voices (Black and white males and females) was used for the meditations.

During the process, the study team also partnered with a graphic designer to develop concepts for a study name and logo to promote study recognition among patients. Additionally, surveys and data collection tools were developed to measure outcomes in a future pilot study testing the MBI.

#### Intervention Development Sessions

Individual intervention development sessions with patients and medical staff were conducted by videoconferencing to obtain feedback about the developed MBI (August–October 2022). Inclusion criteria for patients and medical staff were the same as the criteria for the formative interviews with two additional requirements. Participants had to have access to a working computer with Internet connection and working microphone so that developed content could be reviewed and feedback could be obtained during the sessions. In addition, patients could not have a previous diagnosis of a mental disorder or current participation in mental health counseling or therapy to mitigate any potential adverse effects while reviewing the mindfulness meditations.

Potentially eligible patients and medical staff were recruited from participants who completed the formative interviews and additional participants were recruited using convenience sampling with assistance from a practicing gastroenterologist. Potentially eligible patients and medical staff were contacted to confirm eligibility, assess interest, and obtain verbal informed consent, which included permission to record the session.

Five patients completed two intervention development sessions. The first session obtained feedback on the initial developed MBI content, and the second session reviewed incorporated revisions based on feedback from the first session. Medical staff completed one session to review the content for medical accuracy.

During the first session that lasted about 90 min, patients suggested revisions to the meet the study team infographic including limiting it to one page, removing the description of the role of study team, and emphasizing who will meet them in the waiting room. Patients also suggested revisions to the CRC screening and mindfulness infographics including reducing and simplifying messages to three or four points, prioritizing the main message, and avoiding words that may invoke a negative emotional response.

During the second session that lasted about an hour, patients reported that the revised infographics were improved and looked credible and professional. Additionally, patients were satisfied with the four recorded meditation voices and suggested that patients should have the option of choosing the voice they wanted. Among the meditation background photographs and audios reviewed, patients preferred a picture of a hammock on a beach and the sound of waves for the audio. Lastly, patients preferred the study name of “For peace of mind. Get Screened” and suggested to add the tagline “A colon screening project” for clarity.

Three medical staff attended one intervention development session that lasted about 15 min and approved the infographics with minor wording changes. For example, the message, “Did you confirm your ride to your colonoscopy?” was revised to be more specific, “Did you confirm your ride home after your colonoscopy?”.

#### Individual Review Sessions

Individual review sessions lasted about 1 h and were conducted by videoconferencing with a convenience sample of age-appropriate community members in February 2023. The sessions were conducted to determine the usability of the links used on the study landing page and to review the MBI content including a draft of the survey. Community members were eligible if they were ages 45–75, were at average-risk for CRC, were able to speak and read English, and had access to a working computer with Internet connection and a working microphone. Community members were excluded if they had an auditory or visual impairment, had a previous diagnosis of a mental disorder, or were currently participating in mental health counseling or therapy.

Potentially eligible participants were contacted to confirm eligibility, assess interest, and obtain verbal informed consent including permission to record the session. The four participating community members thought the hammock on the beach photograph and wave audio were well-suited for the mindfulness meditations. Additionally, participants reported that the survey questions were straightforward and non-intrusive, and it was easy to answer questions using a computer or mobile platform.

## Results

### Participant Demographic Characteristics

Participant demographic characteristics for the interviews and sessions are provided in Table 1.

**Table 1:** Demographic characteristics of participants

	Formative interviews		Intervention development sessions		Individual review sessions
	Patients	Medical Staff	Patients	Medical staff	Adults
	*N* = 12	*N* = 6	*N* = 5	*N* = 3	*N* = 4
Age: average years (SD)	52.5 (5.5)	43.0 (12.5)	51.0 (4.2)	46.7 (15.8)	53.8 (4.2)
Gender Male Female	6 6	- 6	3 2	1 2	2 2
Ethnicity/race Non-Hispanic African-American/Black Non-Hispanic White Non-Hispanic Other	8 3 1	3 3 -	3 2 -	- 2 1	- 4 -
Health insurance Yes	12	-	5	-	4
Education Less than high school High school/GED Four-year college Graduate/professional	1 4 2 5	- - 3 3	- 1 - 4	- - 1 2	- - 3 1

*SD* standard deviation, *GED* General Educational Development

### Themes

Two main themes emerged from the study. First, both patients and medical staff reported that average-risk patients have pre-procedural anxiety before a first-time screening colonoscopy. This anxiety emanates from hearing about other peoples’ CRC screening experiences, and their worry about the bowel prep process, the colonoscopy, and the test results. The second theme to emerge is the importance of using stakeholder-engaged strategies including both patients and medical staff in an iterative process so that the developed intervention will be acceptable to the priority population, ensure medical accuracy, and to confirm that the intervention will not interfere with staff workflow.

### Study Name and Logo

**Fig. 1:**
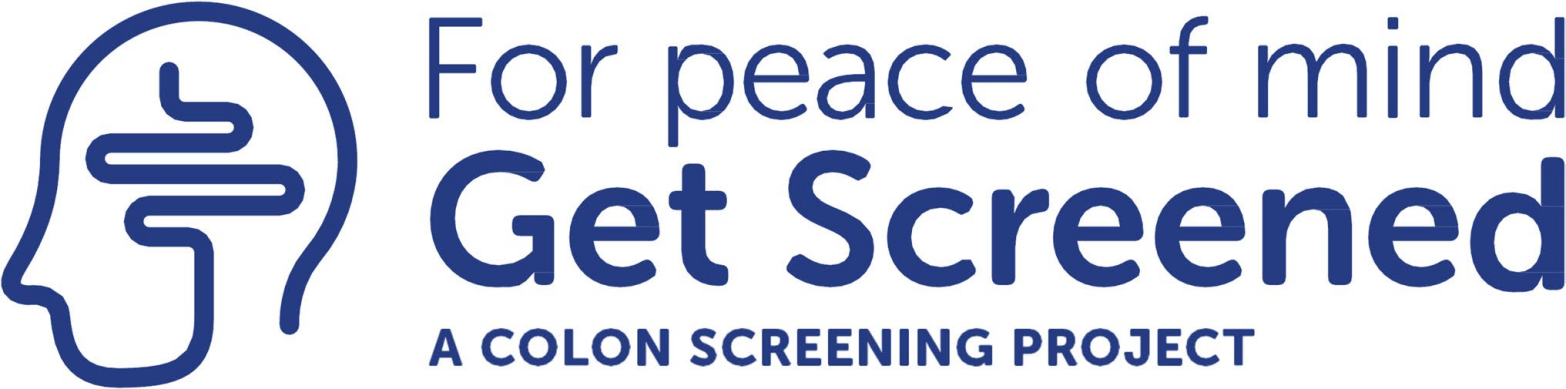
Mindfulness-based intervention study name and logo

The finalized study logo includes “For peace of mind. Get Screened” and the patient suggested tagline “A colon screening project” (Fig. 1).

### Infographics

The revised meet the study team infographic included photos of the study team to establish credibility and highlights the team member who will meet patients in the waiting room before their colonoscopy. The daily CRC screening infographics included messages guided by the PMT constructs and examples are listed in Table 2. Mindfulness infographic messages addressed common misconceptions of mindfulness (e.g., mindfulness is not a religion or religious practice) and benefits of mindfulness practice (e.g., mindfulness can lower stress and improve your bowel prep experience).

**Table 2:** Examples of theoretically based infographic CRC and CRC screening messages

Protection motivation theory constructs	Messages
Threat appraisal	
Perceived severity	Colorectal cancer is serious, and you should not delay screening
Perceived vulnerability	Men and women are at risk for developing colorectal cancer
Intrinsic/extrinsic rewards	A colonoscopy may save your life and remember you are important to your family and friends
Coping appraisal	
Response efficacy	A colonoscopy may prevent colorectal cancer or detect it at an early stage when treatment is more successful
Self-efficacy	You can do this! [complete bowel prep and colonoscopy]
Response costs	Set alarms for taking your bowel prep

*CRC* colorectal cancer

### Mindfulness Meditations

Daily mindfulness meditations included a brief introduction followed by statements guided by MAT constructs of attention monitoring (e.g., now direct your attention to your stomach and to your belly, what do you feel?) and acceptance (e.g., whatever feeling you have is correct, there are no wrong answers, just notice what your feeling is).

## Discussion

The themes that emerged from this study were important when developing the brief online MBI to reduce anxiety among average-risk patients undergoing a first-time colonoscopy. Patient anxiety before a colonoscopy is consistent with studies that have identified it as a common patient barrier because of the bowel prep process, the procedure’s invasiveness, and concern about test results [7, 8]. Although this patient-level barrier has been previously reported, it was key to discuss anxiety with patients and medical staff to ensure that the developed daily messages and meditations included in the MBI would be appropriate, accurate, and potentially effective. The iterative process used and the importance of including stakeholders in the development of interventions has also been previously reported [24, 25]. The codesign process used to develop the MBI was central to include content that is appropriate, acceptable, medically accurate, and to ensure feasibility by not disrupting workflow in a medical setting.

Although previous research supports the use of MBIs to improve the mental health of cancer patients and survivors [19], currently, there is a lack of evidence that MBIs may reduce anxiety prior to cancer screening. Furthermore, our understanding of the effectiveness of MBIs is limited by the lack of transparency and mindfulness theory incorporated into MBI design, implementation, and evaluation [20].

Study strengths include the reporting of the methodology used to develop the MBI and that the intervention materials were guided by behavioral and mindfulness theories. Additionally, we used an iterative process that included patients, medical staff, community members, and study team members. By using this process, it provides the greatest likelihood that the MBI will be feasible and acceptable when tested in a future pilot study.

Study limitations include the small number of stakeholders from one healthcare system located in one geographic region who provided input into the intervention development. Although they may not reflect the viewpoints of other patients or medical staff, data saturation was achieved during the process. Another limitation was that the development process was planned to be conducted in-person; however, because of the COVID-19 pandemic, the sessions were completed by phone or videoconferencing. It is uncertain if this change in the intervention development process may have affected the study.

## Conclusions

We used an iterative stakeholder-engaged process to develop a theory-guided MBI to reduce pre-colonoscopy anxiety among average-risk patients. If efficacious, the MBI has the potential to reduce pre-procedural anxiety which may improve patient behaviors (e.g., bowel prep adherence and quality), patient satisfaction, and clinic workflow by reducing cancellation/no-shows, the amount of sedation required, and procedural time.

This manuscript is part of Brent Emerson’s (principal author) dissertation research.

## Declarations

### Identifiable Data

I confirm all patient/personal identifiers have been removed or disguised so the patient/person(s) described are not identifiable and cannot be identified through the details of the story.
